# Advanced practice physiotherapy care in emergency departments for patients with musculoskeletal disorders: a pragmatic cluster randomized controlled trial and cost analysis

**DOI:** 10.1186/s13063-023-07100-x

**Published:** 2023-02-06

**Authors:** E. Matifat, E. Berger Pelletier, R. Brison, L. J. Hébert, J.-S. Roy, L. Woodhouse, S. Berthelot, R. Daoust, M.-J. Sirois, R. Booth, R. Gagnon, J. Miller, Y. Tousignant-Laflamme, M. Emond, K. Perreault, F. Desmeules

**Affiliations:** 1 Maisonneuve-Rosemont Hospital Research Center, University of Montreal Affiliated Research Center, Montréal, Québec Canada; 2 Faculty of Medicine, Université Laval Québec, Québec, Canada; 3 Department of Emergency Medicine, Queen’s University, Kingston, Ontario Canada; 4 Center for Interdisciplinary Research in Rehabilitation and Social Integration (Cirris), Québec, Canada; 5 Department of Rehabilitation, Faculty of Medicine, Laval University, Québec, Québec, Canada; 6 Tufts University School of Medicine, Public Health and Community Medicine, Boston, Arizona USA; 7 Department of Family Medicine and Emergency Medicine, Faculty of Medicine, Laval University, Québec, Québec, Canada; 8 School of Rehabilitation Therapy, Faculty of Health Sciences, Queen’s University, Kingston, Ontario Canada; 9 School of Rehabilitation, Faculty of Medicine and Health Sciences, Sherbrooke University, Sherbrooke, Québec, Canada; 10 School of Rehabilitation, Faculty of Medicine, University of Montréal, Montréal, Québec, Canada

**Keywords:** Advanced practice physiotherapy, Physiotherapist, Emergency department, Musculoskeletal disorders, Primary care, Model of care, Economic analysis, Cost analysis

## Abstract

**Background:**

Advanced practice physiotherapy (APP) models of care where physiotherapists are primary contact emergency department (ED) providers are promising models of care to improve access, alleviate physicians’ burden, and offer efficient centered patient care for patients with minor musculoskeletal disorders (MSKD).

**Objectives:**

To compare the effectiveness of an advanced practice physiotherapist (APPT)-led model of care with usual ED physician care for persons presenting with a minor MSKD, in terms of patient-related outcomes, health care resources utilization, and health care costs.

**Methods:**

This trial is a multicenter stepped-wedge cluster randomized controlled trial (RCT) with a cost analysis. Six Canadian EDs (clusters) will be randomized to a treatment sequence where patients will either be managed by an ED APPT or receive usual ED physician care. Seven hundred forty-four adults with a minor MSKD will be recruited. The main outcome measure will be the Brief Pain Inventory Questionnaire. Secondary measures will include validated self-reported disability questionnaires, the EQ-5D-5L, and other health care utilization outcomes such as prescription of imaging tests and medication. Adverse events and re-visits to the ED for the same complaint will also be monitored. Health care costs will be measured from the perspective of the public health care system using time-driven activity-based costing. Outcomes will be collected at inclusion, at ED discharge, and at 4, 12, and 26 weeks following the initial ED visit. Per-protocol and intention-to-treat analyses will be performed using linear mixed models with a random effect for cluster and fixed effect for time.

**Discussion:**

MSKD have a significant impact on health care systems. By providing innovative efficient pathways to access care, APP models of care could help relieve pressure in EDs while providing efficient care for adults with MSKD.

**Trial registration:**

ClinicalTrials.govNCT05545917. Registered on September 19, 2022

## Administrative information

**Table:** 

Title {1}	Advanced practice physiotherapy care in emergency departments for patients with musculoskeletal disorders: a pragmatic cluster randomized controlled trial and cost analysis
Trial registration {2a and 2b}.	ClinicalTrials.gov identifier: NCT05545917
Protocol version {3}	Version16, issue date: October 10, 2021
Funding {4}	This research is funded by the Canadian Institutes of Health Research (CIHR) (202010PJT-451693-CIB-CFCC-130299).
Author details {5a}	Matifat, E.: Maisonneuve-Rosemont Hospital Research Center, University of Montreal Affiliated Research Center, Montréal, Québec, Canada. Berger Pelletier, E.: Faculty of Medicine, Université Laval Québec, Québec, Canada Brison, R.: Department of Emergency Medicine, Queen’s University, Kingston, Ontario, Canada. Hébert, L.J.: Center for Interdisciplinary Research in Rehabilitation and Social Integration (Cirris); Department of Rehabilitation, Faculty of Medicine, Laval University, Québec, Québec, Canada. Roy, J.-S.: Center for Interdisciplinary Research in Rehabilitation and Social Integration (Cirris); Department of Rehabilitation, Faculty of Medicine, Université Laval Québec, Québec, Canada. Woodhouse, L.: Tufts University School of Medicine, Public Health and Community Medicine, Arizona, USA. Berthelot, S.: Department of Family Medicine and Emergency Medicine, Faculty of Medicine, Laval University, Québec, Québec, Canada. Daoust, R.: Department of Family Medicine and Emergency Medicine, Faculty of Medicine, Montreal University, Montréal, Québec, Canada. Sirois, M.-J. : Department of Rehabilitation, Faculty of Medicine, Laval University, Québec, Québec, Canada. Booth, R.: School of Rehabilitation Therapy, Faculty of Health Sciences, Queen’s University, Kingston, Ontario, Canada. Gagnon, R.: Center for Interdisciplinary Research in Rehabilitation and Social Integration (CIRRIS), Québec, Québec, Canada; Department of Rehabilitation, Faculty of Medicine, Laval University, Québec, Québec, Canada. Miller, J. : School of Rehabilitation Therapy, Faculty of Health Sciences, Queen’s University, Kingston, Ontario, Canada. Tousignant-Laflamme, Y. : School of Rehabilitation, Faculty of Medicine and Health Sciences, Sherbrooke University, Sherbrooke, Québec, Canada. Emond, M. : Department of Family Medicine and Emergency Medicine, Faculty of Medicine, Laval University, Québec, Québec, Canada. Perreault, K.: Center for Interdisciplinary Research in Rehabilitation and Social Integration (Cirris), Québec, Québec, Canada; Department of Rehabilitation, Faculty of Medicine, Laval University, Québec, Québec, Canada. Desmeules, F.: Maisonneuve-Rosemont Hospital Research Center, University of Montreal Affiliated Research Center, Montréal, Québec, Canada; School of Rehabilitation, Faculty of Medicine, University of Montréal, Montréal, Québec, Canada.
Name and contact information for the trial sponsor {5b}	N/A, no sponsor
Role of sponsor {5c}	N/A, no sponsor

## Introduction

### Background and rationale {6a}

Overcrowding in emergency departments (ED) is one of the biggest concerns in several health care systems. This challenge will worsen in years to come given the aging populations, the increasing prevalence of chronic disorders, and physician shortages [1, 2]. Each year, there are almost 16 million visits made to Canadian EDs [3], and recent reports indicate that Canada is among the countries with the longest ED waiting times [3, 4]. Musculoskeletal disorders (MSKD) have been identified as one of the most prevalent disorders why persons consult EDs [2, 5–7]. Patients presenting to EDs with MSKD, such as tendinopathy, back pain, sprains, and osteoarthritis, could represent at least 25% of all visits [8, 9]. MSKD are also one of the most disabling and costly non-fatal health disorders [10, 11]. Currently, MSKD affect 11 million Canadians per year, and this number is expected to increase [12]. In Canada, MSKD are estimated to cost over 25 billion dollars per year [13].

Traditionally, ED physicians are the primary contact practitioners who manage patients with MSKD, although a high proportion of physicians report limited knowledge and confidence in taking charge of these patients [8, 14, 15]. Furthermore, these tasks deter ED physicians from managing patients with more serious conditions. Several initiatives have been implemented worldwide to improve not only access, but also quality of care for patients with MSKD [16]. New collaborative models of care integrating physiotherapists (PTs) in roles referred to as advanced practice physiotherapy (APP) have been emerging in various settings, such as orthopedic clinics and EDs. PTs in Canada have long had direct access [17] to patients for traditional rehabilitation as they have extensive expertise in MSKD care [18]. Traditionally, PTs provided care in EDs only after physicians assessed patients and made a referral for physiotherapy care. APP models of care in EDs allow for collaborative practice with physicians that is aimed to benefit ED performance, patient care, and outcomes, as well as health care resource usage. In these roles, PTs can also triage patients and, in some jurisdictions, order medical imaging or prescribe/deprescribe certain medications, in addition to providing recommended non-pharmacologic interventions for the management of MSKD [19]. Studies have shown that APP care for MSKD, such as low back pain, significantly decreases opioid prescriptions and long-term use [20–22]. In countries such as Australia and the United Kingdom (UK), integrating APP care in the ED has shown to improve wait times and quality of care for patients with MSKD [23], with high patient and ED physician satisfaction [24–28]. While models of APP care in the ED are emerging globally, none of these models has been evaluated in Canada, where these models are also emerging.

### Objectives {7}

Thus, the aim of our project is to evaluate the impact of an APP-led model of care compared to usual physician ED care for persons presenting with minor MSKD. The specific objectives are:

1. To compare the effectiveness of both types of care in terms of patient-related outcomes for patients with minor MSKD in six Canadian EDs, at discharge from the ED and 4, 12, and 26 weeks after the ED consultation, on the following outcomes:
  - Primary outcome: the Brief Pain Inventory (BPI) – Interference of pain on function subscale
  - Secondary outcomes: pain intensity, disability, patient satisfaction with care, and adverse events.
2. To compare health care resource utilization between both types of care at ED discharge and at 4, 12, and 26 weeks after the initial consultation, including proportion of ED return visits for the same complaint, medication prescriptions, prescriptions for imaging and laboratory tests, and referral to other health care providers
3. To compare both types of care in terms of health care costs from the public payer’s perspective and perform a cost-utility analysis, at ED discharge and at 4, 12, and 26 weeks after the initial consultation
4. To compare wait to initial assessment and ED length of stay between both types of care for the evaluation and treatment of participants during their ED visit

We hypothesize that integrating APP-led care in the ED will be at least as effective as usual ED physician care for patients with a MSKD, in terms of patient-related and health care resource utilization-related outcomes. Furthermore, health care costs will be lower in the APP-led care arm.

### Trial design {8}

This project is a multicenter pragmatic stepped-wedge (3 steps with 4 periods of 6 weeks each) cluster RCT with a cost analysis conducted in six Canadian EDs.

## Methods: participants, interventions, and outcomes

### Study setting {9}

Patients will be recruited from the following six Canadian EDs: Maisonneuve-Rosemont Hospital (MRH) (CIUSSS-de-l’Est-de-l’Ile-de-Montréal, Montréal, Québec), Centre Hospitalier Universitaire de l’Université Laval (CHUL) and Enfant-Jésus Hospital (EJH) (CHU de Québec, Québec), Hotel-Dieu Hospital (Kingston Health Sciences Center-KHSC, Kingston, Ontario), Hôtel-Dieu Hospital (CIUSSS-de-l’Estrie-CHUS, Sherbrooke, Québec), and Rockyview General Hospital (Calgary, Alberta).

### Eligibility criteria {10}

The inclusion criteria are (1) patients presenting with complaints related to minor MSKD (e.g., back pain, joint sprain, osteoarthritis, muscle pain, or tendinopathy) triaged as level 3, 4, or 5 on the Canadian Triage and Acuity Scale (CTAS); (2) aged 18 years or more; (3) legally able to consent; (4) able to understand/speak French or English; and (5) be a beneficiary of a provincial universal health insurance coverage.

The exclusion criteria are (1) having injury resulting from major trauma (e.g., high-velocity trauma or major motor vehicle accident); (2) presenting a major musculoskeletal injury (e.g., open fractures, unreduced dislocations, open wounds, or a condition that needs an urgent surgical intervention); (3) presenting a red flag (e.g., progressive neurological deficits or infection-related symptoms); (4) consulting for a diagnosed inflammatory arthritis or other active/unstable non-musculoskeletal condition (e.g., pulmonary, cardiac, digestive or psychiatric condition); and (5) consulting for a work-related MSKD eligible for workers’ compensation benefits.

### Who will take informed consent? {26a}

Research professionals in each site will explain the study, including the purpose and the required implication by participants, to eligible patients and will answer any remaining questions. Research professionals will then obtain written informed consent from patients who agree to enroll in this study. Information and consent forms will be available in both French and English, depending on the participants’ preferred language.

### Additional consent provisions for collection and use of participant data and biological specimens {26b}

N/A, no ancillary studies

### Interventions

#### Explanation for the choice of comparators {6b}

In the Canadian health care system, patients presenting to EDs will receive the usual care from an ED physician. As this is the most prevalent model of care for patients with MSKD, this will be used as our control arm.

#### Intervention description {11a}

The experimental arm will receive APP-led care. Patients will be independently managed (assessment and intervention) by a PT (*n* = 12, two per site). The PT will make a diagnosis and initiate an intervention plan (e.g., education and exercise). If relevant, the PT will make recommendations for medical imaging tests or medication. PTs will also recommend the proper discharge from the ED, such as hospitalization, discharge without medical consultation/follow-up, or discharge with a medical follow-up or rehabilitation in an outpatient setting. To ensure that these medical recommendations made by PTs in respect of the current professional legislations, an independent ED physician, not participating in the usual care arm, will be present in the ED and will be available to ensure the required medical care according to the PTs intervention plan (e.g., prescribe medication/tests/referrals) if needed. In the event that modification to the plan is found necessary (i.e., only if found unsafe for the patient) by the independent ED physician, this will be recorded and secondary analyses including only participants with no plan modifications will be performed.

The control arm will receive the usual ED physician care delivered by an ED physician. This will include independent assessment, treatment, and discharge by an ED physician with no physiotherapy within the ED but a physician’s referral to outpatient physiotherapy or other professionals/medical specialists will be possible. Overall care offered by the ED physician will not be standardized but will be systematically documented.

**Fig. 1:**
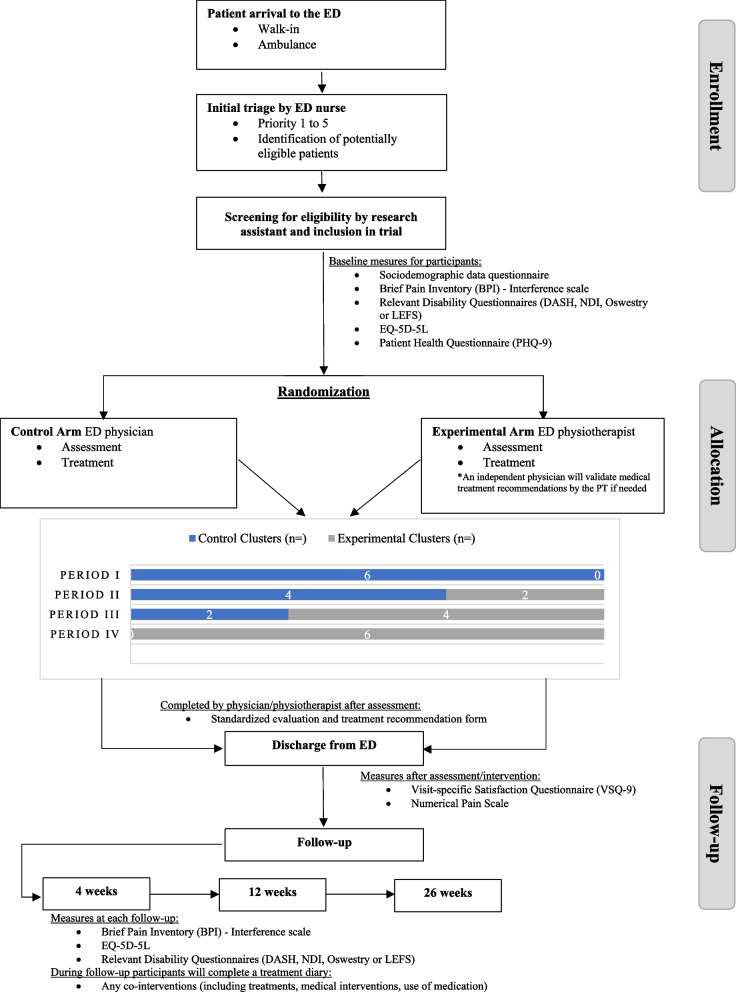
Trial flow chart

Participants in both arms presenting a non-MSKD condition after assessment will be excluded from the study. The proportion of excluded participants per group, as well as the reasons for exclusion, will be systematically collected and analyzed (Fig. 1).

#### Criteria for discontinuing or modifying allocated interventions {11b}

No specific criteria for modifying allocated interventions were defined since this is a punctual intervention. However, participants in both arms presenting a non-MSKD condition after assessment will be excluded from the study and will receive usual ED physician care outside the trial according to their condition.

#### Strategies to improve adherence to interventions {11c}

This pragmatic trial will evaluate the impact of an assessment and intervention offered only within the ED either by a PT or a physician.

#### Relevant concomitant care permitted or prohibited during trial {11d}

There will be no restriction in terms of care during this trial. Participants will be asked to complete a treatment diary regarding compliance with ED treatments and any health services or interventions they sought for their initial problem during the follow-up period.

#### Provisions for post-trial care {30}

The main potential risk in this trial is a diagnostic error by PTs or physicians. Any adverse events will be documented within the current study, and we will ensure study participants receive the appropriate care if needed.

### Outcomes {12}

#### Baseline data collection

At trial inclusion, eligible participants will answer a series of questions covering socio-demographic characteristics, such as age, sex, gender, education level, household income, and living status. Clinical variables such as anthropometric data, affected body area, reason for consultation, CTAS level, duration of symptoms, type of disorder (traumatic/atraumatic), use of walking aids, and presence of any comorbidities will also be documented. Participants will also respond to the Patient Health Questionnaire (PHQ-9), a multipurpose instrument for screening and measuring depression [29, 30].

#### Primary outcome measure

The main outcome measure will be the Brief Pain Inventory–Short Form, Pain Interference Scale (BPI), and the primary endpoint at 4 weeks after discharge. The BPI is a self-administered questionnaire that includes seven items where the patient is asked to rate the impact of pain on various functional activities (pain interference scale) using a 10-point scale. The BPI is valid, reliable, and responsive to change in MSKD populations [31–34] and is suited for electronic administration [31, 35, 36].

#### Other patient-related outcomes

In order to further assess the impact of MSKD on function, participants will complete self-reported validated and reliable disability questionnaires relevant to the affected body area. Depending on the affected body area(s), participants will complete one or more of the following questionnaires: Neck Disability Index (NDI) for neck-related disorders [37], Oswestry Disability Index (ODI) for back-related disorders [38], short version of the Disability of the Arm, Shoulder and Hand (Quick DASH) for upper limb related disorders [39, 40], or Lower Extremity Functional Scale (LEFS) questionnaire for lower limb related disorders [41, 42]. To assess satisfaction with care, patients will be asked to complete a modified validated version of the 9-item visit-specific satisfaction questionnaire (VSQ-9) at initial discharge [43–46]. Participants will be informed that these answers will remain confidential and will not be communicated to the ED providers.

#### Health care resource utilization outcomes

The ED physician or PT will complete a standardized form following their assessment and interventions, indicating diagnoses, requests for additional medical imaging or laboratory tests (if relevant), treatment plan (e.g., conservative treatment options, medication or physiotherapy care), discharge plan, and referral to other professional or medical specialists, if relevant. Providers in both arms will have access to the patient’s full medical record. Proportion of medical imaging tests or other laboratory tests, drug prescriptions/recommendations for analgesics and for opioids, and referral to other providers after initial assessment will be recorded with this form and confirmed with the participants’ medical file. Any adverse events, re-visits to the ED for the same complaint, and any incidents associated with care will be questioned at each time point during follow-up and completed by consulting patients’ medical files.

#### Health care costs

The EQ-5D-5L will be the primary outcome measure for the cost analysis outcomes. It is a generic health-related quality of life questionnaire for which validity has been established. It has been widely used in economic evaluations, including in trials with patients suffering from MSKD and in our pilot RCT [44]. Time-driven activity-based costing (TDABC) will be used to measure the costs of all resources (personnel, consumables, overhead, etc.) utilized during the ED visit and any episode of care during follow-up. TDABC combines process mapping and resource-level costing and has been used to measure health care costs around the world [47–54]. The overarching objective of this methodology is to calculate the costs of all resources (personnel including PT and physician salaries, consumables, overhead, etc.) consumed as a patient moves along a care pathway, which is determined via a collaboration between clinical and administrative staff and each step of the pathway represents direct and indirect resources consumed when providing patient care. The care pathway in this case includes the ED visit and any episode of care during follow-up visits. The TDABC methodology will be applied at the six different sites and hence allowing us to map and compare the care pathway used in all the different sites and potentially compare them to outcomes achieved using the EQ-5D-5L.

#### Wait to initial assessment and ED length of stay measures

Wait in the ED, which is defined as the time between the arrival in the ED and the initial evaluation by a physician or a PT will be recorded. The total length of stay in the ED will also be recorded in minutes by research professionals onsite.

#### Follow-up measures

The questionnaires will be sent by email using the Research Electronic Data Capture (REDCap™) platform or completed over the phone, depending on patient preference. All included questionnaires have been validated for use electronically or over the phone. REDCap is a secure web platform. In order to limit loss to follow-up, we will validate with participants at each time point their contact information and preferred means of communication. Participants will also be asked to complete a treatment diary regarding compliance with ED treatments and any health services or interventions they sought for their initial ED visit during the follow-up period (Fig. 1, Table 1)

**Table 1:** Time schedule of enrollment, interventions, and assessments

	Study period					
	Enrolment	Allocation	Post-allocation			Close out
**Time point**	***−t***_**1**_	**0**	***t***_**1*(post-eval)***_	***t***_**2*(4 weeks)***_	***t***_**3*(12 weeks)***_	***t***_**4*(26 weeks)***_
**Enrollment**						
**Eligibility screen**	**X**					
**Informed consent**	**X**					
**Sociodemographic data**	**X**					
**Allocation**		**Stepped-wedge design: randomization according to time periods across all sites (3 steps of 6 weeks periods—2 sites switching to experimental arm at each step)**				
**Interventions**						
**Usual ED physician care**						
**PT-led APP care**						
**Assessments**						
**BPI**	**X**			**X**	**X**	**X**
**Disability questionnaires (NDI, ODI, LEFS, quickDASH)**	**X**			**X**	**X**	**X**
**EQ-5D-5L**	**X**			**X**	**X**	**X**
**PHQ-9**	**X**					
**Numerical Pain Scale**			**X**			
**Standardized evaluation form (PT/MD)**			**X**			
**VSQ-9**			**X**			
**Health care resources utilization and health care costs:** **Treatment diary** **Patients’ medical file**				**X**	**X**	**X**

*ED* emergency department, *APP* advanced practice physiotherapy, *BPI* Brief Pain Inventory, *NDI* Neck Disability Index, *ODI* Oswestry Disability Index, *LEFS* Lower Extremity Functional Scale, *DASH* Disabilities of the Arm, Shoulder and Hand, *PHQ-9* Patient Health Questionnaire-9, *VSQ-9* Visit-Specific Questionnaire-9

### Participant timeline {13}

The participant timeline is presented in Table 1.

### Sample size {14}

The main hypothesis is that APP-led care will be at least as effective as usual ED physician care based on the results of our literature review [16] and ED studies [43, 44]. The sample size required is based on a target effect size of 0.35, using the primary outcome measure, the BPI short form, with a two-sided significance level of 0.05 and a power of 0.80. An intra-cluster correlation coefficient of 0.004 was assumed based on a prior cluster RCT that also used the BPI short form as a primary outcome [55]. The calculation further assumed 6 clusters with an exchangeable correlation structure within clusters, a total of 3 sequences (steps), and two clusters changing treatment per sequence. With these assumptions, the trial will be adequately powered if 27 individuals are accrued per cluster per sequence. Hence, we will recruit 31 patients per site per sequence, accounting for a potential loss to follow-up of 15%. The total sample of 744 participants (124 per site) will provide an adequate sample size to meet our objectives.

### Recruitment {15}

All new patients presenting to participating EDs and triaged by nurses (based on the usual triage process used in each ED) as having a possible MSKD will be considered for this study. In addition, a research professional in each site will screen the triage lists to identify any other potential participants for the trial and ensure none are omitted. Eligibility of ED patients will subsequently be assessed regarding the inclusion and exclusion criteria during an in-person interview with a research professional and review of medical records. Research professionals will explain the study to eligible patients and obtain their informed consent.

For patients who decline participation in the project, demographic data, such as age, sex, reason for consultation, and reason to refuse to participate, will be collected to calculate participation proportions and establish comparisons between participants and non-participants.

### Assignment of interventions: allocation

#### Sequence generation {16a}

All eligible and consenting sites will be randomly assigned to a sequence of models of care (APP-led or usual). All sites will begin the trial with a baseline period where no site is in the intervention arm. Then, during 6-week intervals, two sites (clusters) will move to the intervention arm with this timing randomized at the outset of the trial. After the third step in the sequence, all six sites will be in the intervention arm.

#### Concealment mechanism {16b}

Sites will be informed at the beginning of the trial of the intervention sequence as this will allow efficient planning of the intervention arm at each ED.

#### Implementation {16c}

Randomization procedures will be performed by an independent research professional not involved in other aspects of the trial.

### Assignment of interventions: blinding

#### Who will be blinded {17a}

Neither participants nor providers will be blinded in this study in the context of this pragmatic RCT. Even if the patient’s perception can be influenced by the type of provider they see, this approach is necessary because of the pragmatic nature of the proposed care model, and it is important that patients know which provider and models of care they are exposed to as this increases the external validity of the current trial. An independent statistician not involved in any other parts of this trial and blinded to the participants’ allocation will conduct the statistical analyses.

#### Procedure for unblinding if needed {17b}

Neither participants nor providers are blinded.

### Data collection and management

#### Plans for assessment and collection of outcomes {18a}

All data will be collected using the REDCap platform. Outcome measures will be collected at inclusion, before ED discharge, and at 4-week, 12-week, and 26-week follow points (Table 1).

#### Plans to promote participant retention and complete follow-up {18b}

In order to limit loss to follow-up, we will validate with participants at each time point their contact information and preferred means of communication.

#### Data management {19}

The databases will be centralized at the coordination center (Maisonneuve-Rosemont Hospital - MRH - Research Center). MRH will store all data captured in REDCap on its own servers. All data is therefore stored and hosted at MRH, and no project information is ever transmitted at any time by REDCap from MRH to any other institution.

#### Confidentiality {27}

Data will be kept in REDCap at the end of the study in a de-identified form using an identification code. Only the investigators will have access to the link key linking codes to individuals. The data will be kept for seven years.

#### Plans for collection, laboratory evaluation, and storage of biological specimens for genetic or molecular analysis in this trial/future use {33}

No biological specimens were collected.

## Statistical methods

### Statistical methods for primary and secondary outcomes {20a}

Descriptive statistics will be used to present the participants’ characteristics. Baseline demographic data will be compared across groups and the participating EDs to establish the comparability of study samples across intervention arms and clusters. If differences are observed, statistical models will be adjusted, and any difference will be considered. Descriptive statistics will be computed for all outcome measures at different measurement times. Per-protocol and intention-to-treat analysis will be performed. Outcomes will be analyzed at the individual level using mixed models as recommended for stepped-wedge designs [56]. A linear mixed model with a random effect for cluster and fixed effects for the intervention status and time period will be used for continuous outcomes including the primary outcome (BPI score). Separate analyses will be conducted on each of the secondary outcomes. In the case of non-normal outcomes, including re-visits and adverse events, a generalized linear mixed-model framework will be used. Time-driven activity-based costing (TDABC) will be the methodology used to capture the costs of providing services to achieve primary and secondary outcomes. TDABC’s micro-costing approach will allow us to precisely determine and differentiate the cost of providing care based on the specific types of patients. Measuring patient outcomes and costs to achieve those outcomes will provide a more rigorous approach to understanding the value of introducing innovative modifications to existing clinical pathways. This approach has the potential to compare costs, outcomes, and clinical pathways within single health care institutions, among regional care centers and across provinces.

### Interim analyses {21b}

Interim analyses will be conducted at the 1-month follow-up to ensure no safety issue is detected and final analyses will be conducted at the end of the trial.

### Methods for additional analyses (e.g., subgroup analyses) {20b}

Secondary analyses will be performed according to the affected body area (upper limb, lower limb, neck, or back) and will consider site recruitment clustering, since these factors can impact care utilization as well as other outcomes measured in the study. The same is also true for sex and gender for which secondary analyses will also be conducted.

### Methods in analysis to handle protocol non-adherence and any statistical methods to handle missing data {20c}

Participants withdrawing from the study and reasons for withdrawal will be analyzed. Characteristics of participants and non-participants will also be compared. Multiple imputation will be used for missing data handling.

### Plans to give access to the full protocol, participant-level data, and statistical code {31c}

The protocol, data, and statistical code that support the findings of this study will be available from the corresponding author, upon reasonable request.

### Oversight and monitoring

#### Composition of the coordinating center and trial steering committee {5d}

The trial steering committee (TSC) will include co-PIs Drs. Desmeules, Perreault, and Émond, with co-Is and assistants hired in each site. They will be at the core of trial management (as described above). The TSC will meet at the beginning of the study to plan/organize, to ensure the study integrates well within each site’s local reality. Twice a year, the TSC will meet to produce an executive summary of the research program. The research team from Maisonneuve-Rosemont Research Center, under Dr. Desmeules’ supervision, will coordinate the study.

#### Composition of the data monitoring committee, its role, and reporting structure {21a}

Since this is a low-risk intervention and not required by the Ethics Committee, there is no external data monitoring committee.

#### Adverse event reporting and harms {22}

The current literature on the safety of ED physiotherapy care with MSKD patients found no increase in adverse events associated with ED physiotherapist-led care [16, 43]. Moreover, physiotherapists will systematically present to an independent ED physician requests for medical imaging and diagnostic tests, medication prescription, or referrals to other providers, to respect medical legislation and ensure safety. Any adverse events will be documented and fully detailed. They will also be communicated to the CIUSSS-de-l’Est-del’Ile-de-Montréal Ethics Committee and reviewed by the co-principal investigators and co-investigators.

#### Frequency and plans for auditing trial conduct {23}

The TSC will meet every month to discuss issues with recruitment, protocol adherence, and follow-up of participants.

#### Plans for communicating important protocol amendments to relevant parties (e.g., trial participants, ethical committees) {25}

If any important protocol amendments are required, the TSC will meet to discuss the relevance of the proposed changes. Ethics committees of all participating sites will be informed of potential changes. Amendments will be undertaken only after approval of all committees.

#### Dissemination plans {31a}

Several stakeholders and knowledge users, such as patients, administrators, health care practitioners, and decision-makers, have been engaged in the entire process from project inception to preparation of this protocol. They will pursue their active involvement until project completion and dissemination of the results. The results of this study will be adapted for presentation and exchange with collaborators in the different trial settings. A set of recommendations based on the results will be developed and shared. The results of this study, with the involvement of our collaborators and knowledge users, will be presented in each participating hospital through formal on-site or distance meetings and symposiums. Other usual means of dissemination will include communications in conferences related to ED and trauma research and practice (e.g., emergency medicine), rehabilitation or health service organization, and scientific publications in peer-reviewed journals. Outcomes will also be shared with stakeholders (medical and patients’ associations and governments) by producing and sharing policy briefs and synthesis documents.

## Discussion

Current evidence shows that an increasing number of MSKD patients turn to EDs for care [57]. Many studies have shown that the current management of MSKD is often not optimal and is associated with poor outcomes [2, 15, 58]. Current training of ED physicians covers the management of serious, complex, and potentially life-threatening disorders. Training for other non-life-threatening disorders such as MSKD is often more limited. Also, current medical management of MSKD is often associated with high rates of opioid prescriptions [58, 59]. Indeed, Canada is struggling with an opioid crisis and many patients presenting to the ED for painful MSKD are discharged with an opioid prescription [60]. Even though opioids should not be the first-line treatment for the management of MSKD [58, 61–63], they represent a significant part of the current medical management of MSKD pain in the ED. Conversely, recommended non-pharmacological approaches [64], such as physiotherapy, are scarcely used. Studies involving PTs as primary care providers for patients presenting with MSKD have shown that early access to care leads to better outcomes as PTs are able to take more time with patients and can provide more complete education to patients and offer self-management strategies [65, 66]. Patients’ satisfaction with MSKD care is often significantly higher with PT led-care compared to physician care [66]. Our team recently completed two studies that serve as a foundation to the current trial. We first assessed the diagnostic concordance as well as management of ED patients with MSKD by ED PTs in comparison with usual ED physician care in two EDs [43]. The results showed that there was a high diagnostic agreement between PTs and ED physicians with no serious or non-MSKD pathologies being missed by PTs and no adverse event recorded. ED PTs were shown to recommend more physiotherapy care for MSKD, in comparison with usual ED medical care. ED physicians prescribed significantly more prescription drugs, including opioids, to manage MSKD in comparison with ED PTs. [43] The second study by our team was a single-blind pilot feasibility RCT conducted in another ED [44]. Although based on per-protocol analysis, patients that received ED PT-led care had significantly lower pain interference and pain intensity levels at 1 and 3 months after the initial ED visit. Lower proportions of prescription medication, including opioids and lower rates of re-visits to the ED, were also observed in comparison with patients receiving usual medical care at 1 month. ED PT-led care was also associated with significantly fewer prescriptions of medical imaging tests [44].

### Strength and limitations

This study is the first multicenter pragmatic trial with a cost analysis assessing APP-led ED care. The trial has been designed by a group of experts with expertise in the management of MSKD, public health, epidemiology, cost analyses, health services, ED medicine, interprofessional collaboration, rehabilitation, and designing and performing RCTs. The stepped-wedge design is an emerging and original methodology that facilitates the evaluation and implementation of new models of care [67].

Although this trial is a pragmatic design, because of its experimental nature, it may not fully represent usual care that APPTs or ED physicians offer as they are aware of the objective of the trial. Moreover, patients are not blinded to providers that will offer care, and it may influence their perceptions. Still, this approach is necessary because of the pragmatic nature of the proposed trial, and it is important that patients know which provider and models of care they are exposed to as this increases the external validity of the current trial. Also, only a limited number of APPTs (12) will be assessing patients within this study across six EDs across Canada.

While APP-led ED models of care are emerging globally, none of these models has been evaluated across Canada where these models are also becoming more prominent. MSKD not only represent a significant economic burden for Canadians, but also have a significant impact on population health, the Canadian workforce, and our health care system. As such, new models of care that result in more efficient care are needed. Given the high consultation rates for MSKD and known overcrowding in EDs, this trial has the potential to improve access and efficiency of ED care.

### Trial status

Recruitment for this trial should start in the fall of 2022 and be completed by the spring of 2023.

## Data Availability

After all analyses have been conducted and results published, the data that support the findings of this trial will be available upon reasonable request.

## Abbreviations

APP: Advanced practice physiotherapy
APPT: Advanced practice physiotherapist
BPI: Brief Pain Inventory
DASH: Disabilities of the Arm, Shoulder and Hand
ED: Emergency department
KT: Knowledge transfer
KU: Knowledge user
LEFS: Lower Extremity Functional Scale
MSKD: Musculoskeletal disorders
NDI: Neck Disability Index
ODI: Oswestry Disability Index
PHQ-9: Patient Health Questionnaire-9
PT: Physiotherapist
RCT: Randomized controlled trial
TSC: Trial steering committee
VSQ-9: Visit-Specific Questionnaire-9
